# EHMTI-0356. Hypnic headache in a patient with sunct: case report

**DOI:** 10.1186/1129-2377-15-S1-J6

**Published:** 2014-09-18

**Authors:** A Granato, J Fantini, N Koscica

**Affiliations:** 1 Neurologic Clinic Headache Centre University of Trieste, Department of Medical Surgical and Health Sciences, Trieste, Italy

## Case description

A 49 year-old healthy woman developed two rare primary headaches.

At the age of 23 she experienced single painful stabs in the left orbital-temporal region lasting few seconds, every 6-7 months. In the subsequent 3 years the single pain attacks became longer in duration, they were accompanied by ipsilateral conjunctival injection and tearing, and frequency increased to 20 episodes/day for 5-10 consecutive days, with pain free intervals of 6-7 months. Brain MRI ruled out secondary headaches. In the following years, active periods reached the duration of 3 consecutive months, with remissions of 1 month. Six years ago, the patient was evaluated in our Headache Centre, a SUNCT (short-lasting unilateral headache with conjunctival injection and tearing) was diagnosed, and lamotrigine 100 mg/day was effectively administered. The pain recurred after therapy discontinuation, so she was forced to continue lamotrigine.

She was pain-free for further 2 years, then she began suffering from a new occipital bilateral pressing pain, moderate, sometimes with nausea, presenting exclusively during sleep and causing awakening. Attacks occurred usually at 4:00-5.30 a.m., lasted about 60 minutes and gradually became near daily. A diagnosis of hypnic headache was made and the patient was treated with caffeine 30 minutes before sleeping, without headache recurrence.

## Conclusions

At the best of our knowledge, this is the first description of SUNCT and hypnic headache occurring in the same patient. The pathogenetic mechanisms of this coexistence are interesting. In this patient the combined specific treatment of both SUNCT and hypnic headache was effective.

No conflict of interest.

